# Examining Public Concerns and Attitudes toward Unfair Events Involving Elderly Travelers during the COVID-19 Pandemic Using Weibo Data

**DOI:** 10.3390/ijerph18041756

**Published:** 2021-02-11

**Authors:** Xinghua Liu, Qian Ye, Ye Li, Jing Fan, Yue Tao

**Affiliations:** 1 The Key Laboratory of Road and Traffic Engineering of the Ministry of Education, Tongji University, Shanghai 201804, China; 1910893@tongji.edu.cn (X.L.); jing.fan@tongji.edu.cn (J.F.); 2031347@tongji.edu.cn (Y.T.); 2 College of Transportation Engineering, Tongji University, Shanghai 201804, China

**Keywords:** COVID-19, elderly traveler, unfair events, public concerns and attitudes, Weibo

## Abstract

The Chinese government has launched a digital health code system to detect people potentially exposed to the coronavirus 2019 (COVID-19) disease and to curb its spread. Citizens are required to show the health code on their smartphones when using public transport. However, many seniors are not allowed to use public transport due to their difficulties in obtaining health codes, leading to widespread debates about these unfair events. Traditionally, public perceptions and attitudes toward such unfair events are investigated using analytical methods based on interviews or questionnaires. This study crawled seven-month messages from Sina Weibo, the Chinese version of Twitter, and developed a hybrid approach integrating term-frequency–inverse-document-frequency, latent Dirichlet allocation, and sentiment classification. Results indicate that a rumor about the unfair treatment of elderly travelers triggered public concerns. Primary subjects of concern were the status quo of elderly travelers, the provision of transport services, and unfair event descriptions. Following the government’s responses, people still had negative attitudes toward transport services, while they became more positive about the status quo of elderly travelers. These findings will guide government authorities to explore new forms of automated social control and to improve transport policies in terms of equity and fairness in future pandemics.

## 1. Introduction

**Figure 1:**
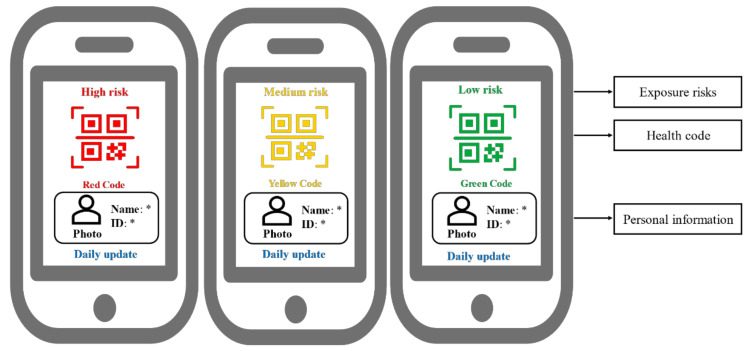
A red-yellow-green system of health codes [4]. The quick response (QR) codes maintain anonymity.

The coronavirus 2019 (COVID-19) disease pandemic has resulted in the death of a substantial portion of the global population [1,2]. The virus responsible for this incident is expected to not resolve completely and may even resurge over time [3]. Thus, the choice between controlling the pandemic and resuming normal public life is a dilemma. To prepare for this new normal, some countries have launched contact tracing apps to identify people who have been potentially exposed to the virus [4]. For example, the Chinese government developed a health code system (see Figure 1) that assigns red, yellow, and green codes to people who are considered high-, medium-, and low-risk for exposure, respectively. Upon confirmation of their code, people who hold green codes are allowed to use public transportation, while people with red or yellow codes are expected to remain in quarantine until obtaining green codes [5]. However, many senior citizens in China are still off-line or do not have smartphones [6], making them unable to obtain the digital health codes that have become an essential tool during the COVID-19 pandemic. This has been a particular problem for elderly people who ride subways or buses in their daily lives. Therefore, elderly people are becoming further disadvantaged due to their inability to access and use information and communication technologies (ITCs) such as health codes [7].

Several unfair events have been reported that are related to elderly travelers without health codes being rejected access to public transportation or indoor places during the pandemic. For example, on 8 August 2020, an elderly traveler who could not show his health code was refused entry to the subway station in Dalian, China, receiving the clicks of six million people on the TikTok platform [8]; on 19 August 2020, an elderly traveler in Harbin, China was refused boarding on the bus by the driver due to not having a health code, which attracted 220 million views on Sina Weibo [9]. These events have sparked extensive public concerns, being regarded as having caused serious damage to the rights of elderly people to travel and go on other activities. Considering the goal of equity and fairness of public policies, transport departments and government authorities need to be fully aware of public concerns and attitudes, especially regarding such unfair events.

Data sources of previous studies on public concerns and attitudes have mainly relied on in-depth interviews, questionnaires, and telephone surveys [10,11,12,13]. Frequently used methods such as qualitative and statistical analysis are used in these studies. As the amount of data obtained using these methods is limited, it is difficult to capture public perceptions comprehensively. Recently, the rapid development of social media has provided a channel for the public to obtain information and share feedback beyond the limitations of space and time. People are more willing to express their opinions on such matters, thereby generating large quantities of data reflecting their real thoughts [14]. Sina Weibo (or Weibo), one of the largest social media sites in China [15], stores massive amounts of information. During the COVID-19 pandemic, such unfair events concerning elderly travelers have sparked heated public discussions on Weibo. For example, as of 14 January 2020, a report about “elderly travelers and health codes” was labeled as a trending hashtag 16 times by Weibo. Meanwhile, automatic data collection techniques such as application programming interface (API) and web crawlers enable the acquisition of large quantities of data from social media. Furthermore, natural language processing (NLP) can be used to understand and manipulate social media data. Therefore, the available social media data and analytical tools provide the potential to capture public concerns and attitudes toward unfair events involving elderly travelers.

Research on transportation issues using social media data has mainly focused on emergency management [16], service satisfaction assessment [17], and human mobility patterns [18]. For example, Zuo et al. concluded that the LDA model can extract emergency events and classify them for both small and large-scale events using Twitter data [16]. Liu et al. designed an NLP approach of a transportation survey using public comments on websites for the appraisal of passenger satisfaction and the service quality of public transportation in Shanghai [17]. Liu et al. used social media data to extract nationwide inter-urban movements and to analyze the underlying patterns of trips and spatial interactions in China [18]. However, there is a lack of research concerning elderly travelers and other vulnerable groups. In particular, the outbreak of COVID-19 has affected the elderly population much more severely than the younger demographic [19]. Most of the elderly population mainly rely on public transport (i.e., subways and buses) for daily movement [20,21]. However, their mobility is compromised by the unavailability of public transportation services during the pandemic. To the best of the authors’ knowledge, there have been few attempts to study the unfair treatment of elderly travelers during the pandemic from the perspective of the general public.

With the help of a dataset collected from Weibo, this study aimed to explore public views and responses to the above-mentioned unfair events concerning elderly travelers without health codes during the COVID-19 pandemic. The study demonstrates the significance of protecting the rights of elderly travelers in any future pandemic. Specifically, we have made the following contributions:

- We collected a dataset of seven-month Weibo messages related to the unfair events involving elderly people during the COVID-19 outbreak. This was done to capture public perceptions on the issue of health code measures through the comprehensive use of our web crawler procedure.
- We developed a hybrid Weibo mining framework integrating the term-frequency–inverse-document-frequency (TF-IDF) algorithm, the latent Dirichlet allocation (LDA) model, and sentiment analysis provided by the Baidu AI platform. This framework was constructed to identify the discussion topics from the general public as well as their attitude toward the unfair events.
- Our findings provide suggestions for government authorities and other transportation stakeholders to make policy decisions on the transportation system by considering equity and fairness for elderly travelers. Moreover, understanding public perceptions toward transportation policies during the pandemic can assist transportation agencies to better prepare for a mobility system for future outbreaks.

The remainder of this paper is organized as follows. Section 2 introduces a hybrid analytical framework consisting of Weibo data collection and three NLP methods. Section 3 presents the results and discussion. Finally, the conclusions and limitations of this study are presented in Section 4, with a note on the scope for future research.

## 2. Methodology

**Figure 2:**
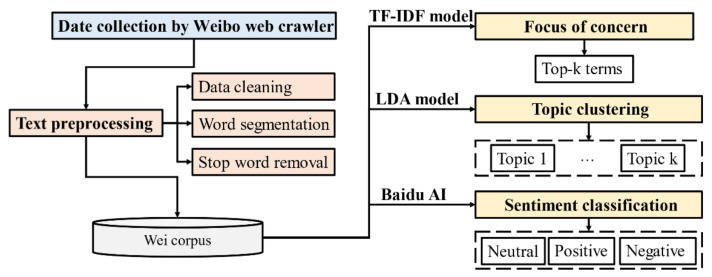
A hybrid framework integrating multiple natural language processing (NLP) methods.

Based on the NLP procedure, a hybrid Weibo mining framework was developed herein to explore public concerns and attitudes [22], as shown in Figure 2. First, we collected social media data using a Weibo web crawler. Second, through data cleaning, word segmentation, and stop-word removal, a Weibo corpus was created. Finally, with the application of the TF-IDF algorithm [23], LDA model [24,25,26], and sentiment classification provided by the Baidu AI platform [27], we determined the focus of public concern, discussion topics, and explored public attitudes.

### 2.1. Data Collection and Pre-Processing

**Figure 3:**
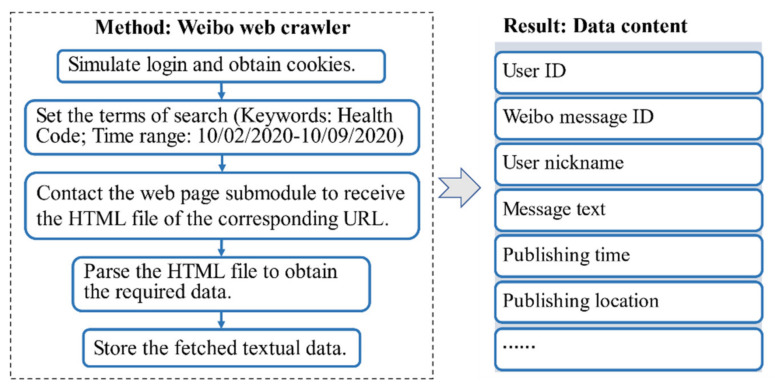
Weibo data collection procedure.

Weibo API [28] and web crawler [29] are two approaches to collect Weibo data. However, because official API has restrictions on data fetching, only a limited amount of Weibo messages can be downloaded. Thus, a Weibo web crawler using Weibo-scraper 1.0.6 [30], a Python package, was designed for data collection. Given the unfair events concerning elderly travelers without health codes, we used “Health Code” as a keyword to search the messages through Weibo. We set the start time for data collection earlier than the date that the health code system was implemented in China (16 February 2020), which was 10 February. After September 2020, there was little discussion of such unfair events that could be observed from the media, although government agencies began to take actions to coordinate between health codes and elderly people. Thus, we set the period of data collection from 10 February 2020 to 10 September 2020. The data included user ID, message ID, user nickname, the content of the message, publishing time, and publishing location, among other information. The data collection procedure is summarized in Figure 3.

Three steps of text preprocessing were used in this study to ensure the accuracy of Weibo messages. The first step was data cleaning, wherein meaningless information, advertisements, and repetitive hashtags were removed. Unlike English sentences, Chinese sentences are continuous and do not contain spaces between words; thus, a Chinese word segmentation toolkit with high efficiency and accuracy, Jieba, was used in the second step. The last step was the removal of stop words, which are functional words without actual meaning such as “of”, “in”, and “the” in English. 

**Figure 4:**
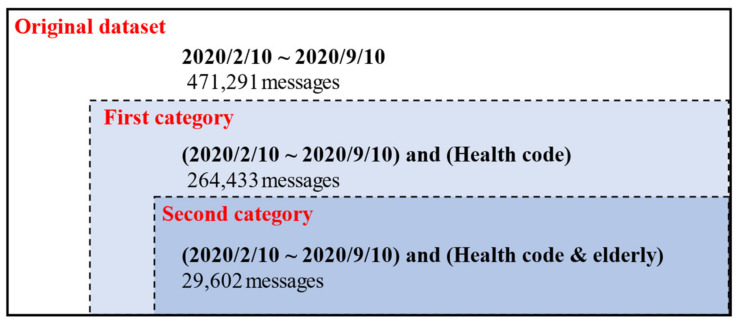
Description of Weibo data in the stages of data collection and preprocessing.

The result of text preprocessing is summarized in Figure 4. The original dataset contains 471,291 rows of Weibo messages mentioning “Health Code”, covering the whole period of data collection. In total, 206,858 rows of data were deleted as noise. Next, we divided the cleaned Weibo data into two categories: the first category contained 264,433 messages mentioning “Health Code” and the second category contained 29,602 messages that mentioned both “the elderly” and “Health Code”. Datasets of the first and second categories were used in further analysis.

### 2.2. Term-Frequency–Inverse-Document-Frequency (TF-IDF) Algorithm

The TF-IDF algorithm was used in this study to filter the most important terms rather than the commonly used words in Weibo messages. The main idea of the TF-IDF algorithm is as follows: if a word is important for a document, it should be repeatedly mentioned in that document but rarely mentioned in other documents [23]. The term frequency $TF_{ij}$ is defined as the number of times the word *i* is mentioned in the document *j*. The larger this value, the more important the word. The inverse document frequency $IDF_{i}$ represents the number of documents in which the word *i* appears at least once; the larger the value, the more common the word. If word *i* is to be considered important for document *j*, then it should have a large$\;TF_{ij}$ and small $IDF_{i}$. Hence, the TF-IDF is defined as Equations (1)–(3):

(1) $$\;TF_{ij}=\frac{n_{i,j}}{\sum_{k}n_{k,j}},$$

(2) $$IDF_{i}=\frac{\left|D\right|}{\left\{j:t_{i}\in d_{j}\right\}+1},$$

(3) $$TF-IDF_{ij}=TF_{ij}\times IDF_{i}=\frac{n_{i,j}}{\sum_{k}n_{k,j}}\times \frac{\left|D\right|}{\left\{j:t_{i}\in d_{i}\right\}+1},$$

where $n_{i,j}$ is the frequency of occurrence of the term 𝑖 in document$\;d_{j};\;\underset{k}{\sum }n_{k,j}\;$is the sum of occurrences of all words in the document$\;d_{j};\;\;\left|D\right|\;$is the total number of documents in a corpus; and $\left\{j:t_{i}\in d_{i}\right\}$ is the number of documents containing the term $t_{i}$.

### 2.3. Latent Dirichlet Allocation (LDA) Topic Model

The LDA model, one of the most popular methods of topic modeling in unsupervised machine learning, is used to identify latent topics penetrating a specific corpus based on the word frequency of a document [24]. A row of Weibo messages can be regarded as a collection of words with an associated probability of being attached to a given topic. Each topic can then be thought of as a probability distribution comprising the words that one would expect to use when discussing that topic [25,26]. The LDA model does not require any prior annotations or labeling of the documents due to its unsupervised features. All the topics are naturally retrieved from the statistical structure of the word data of the document [31]. To avoid subjectivity in the classification process and discover the maximal number of distinct topics, we clustered all the Weibo messages into 26 probabilistic topics by intuitive experience and manually check. Thereafter, through observations of similarities in keywords, several topics were manually merged.

### 2.4. Text Sentiment Analysis

Sentiment analysis aims to classify the sentiment of a given text based on two or more classes and then to discover the tendencies of people’s attitudes. Recently, the Baidu AI platform, an open-source NLP tool in the Chinese language provided by Baidu Corporation, has been commonly applied in practical business analysis. With a high generalization ability, the application of the Baidu AI platform maintains high accuracy for relatively long Chinese sentences. In terms of sentiment orientation analysis, its accuracy rate can exceed 95% [27]. We chose its sentiment classification module in this study to automatically judge the sentiment polarities for each message and report the corresponding confidence degree. The emotional polarity consists of positive, negative, and neutral emotions. The results of sentiment classification help to analyze public attitudes toward unfair events and track their shifts or changes over time.

## 3. Results and Discussion

### 3.1. Temporal Trends in the Number of Weibo Messages

**Figure 5:**
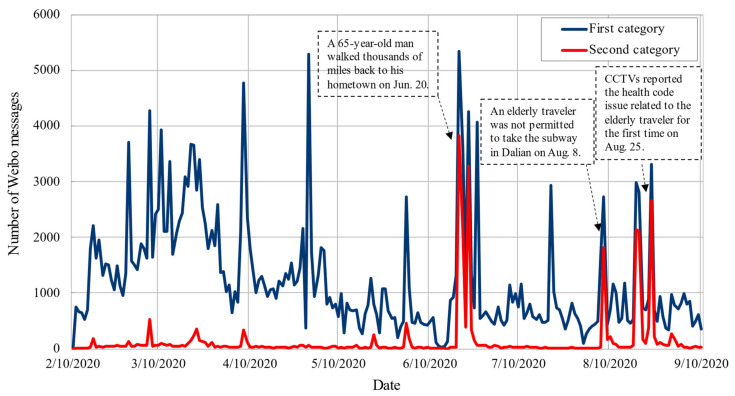
Temporal patterns of the volume of Weibo messages by date.

**Figure 6:**
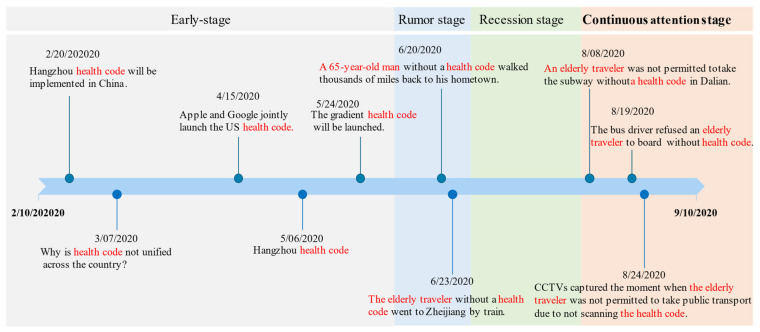
Trending hashtags on health code on Weibo from 10 February 2020 to 10 September 2020.

Figure 5 shows the temporal trends of Weibo messages coming from the two categorized datasets (recall Section 2). Some key events are labeled in the figure including the reported news and the government’s official response. To better explain this temporal pattern, we also filtered the trending hashtags related to health code from the dataset (see Figure 6). 

Figure 5 shows that the number of messages in the second category was less in the period before 20 June compared with the first category, which indicates that the general public did not pay much attention to older people before the first reported unfair events on 20 June. Based on the trending hashtags presented in Figure 6, it seems that the general public was mainly concerned with the importance and promotion of health codes for pandemic prevention. On 20 June, a report of a rumor was published about an elderly citizen walking thousands of miles back to his hometown in Zhejiang Province due to the lack of health codes [32]. This event triggered heated public discussions on Weibo; additionally, it was believed to be a key turning point for promoting public concerns regarding elderly travelers. However, it was proven to be fake news on 23 June [33], which soon dampened public enthusiasm for discussion. The number of Weibo messages experienced a sudden drop and then remained relatively low throughout July. In August, several unfair events were reported such as “elderly person without a health code was refused admission to the subway system” and “elderly person without a health code was refused entry onto a bus by a bus driver”, causing heated public discussions on elderly travelers. The number of messages increased rapidly on 8 August and 19 August. The number of messages peaked when China Central Television (CCTV), a Chinese state media, broadcast a series of news programs focusing on the loss of mobility of elderly people during the pandemic.

During the pandemic, people increased their social media usage [34]. Additionally, people are more likely to be influenced by sensational information and rumors that are quickly spread together with valuable information through the Internet and thus become emotionally anxious during the outbreak. Therefore, policymakers should realize the importance of capturing timely, accurate, and reliable information while suppressing rumors [35]; social media can be a convenient platform for them to understand public demands and better respond when a crisis occurs [36]. 

As shown in Figure 6, we divided the seven months into four stages: early stage (10 February–20 June), rumor stage (20–25 June), recession stage (25 June–1 August), and continuous attention stage (1 August–10 September). The number of messages about elderly people in the early stage and recession stage was relatively small, and the most discussed topics were fake news in the rumor stage, which indicates that these stages are not conducive to exploring real public concerns and attitudes. In the continuous attention stage, the number of messages remained at a relatively high level, and the public discussion was basically about real reports. Therefore, this study chose the continuous attention stage for further analysis. There was a total of 10,505 Weibo messages related to the unfair events regarding elderly travelers and health codes, accounting for 35% of the total number of messages of the second category.

### 3.2. Focus of Public Concern

**Figure 7:**
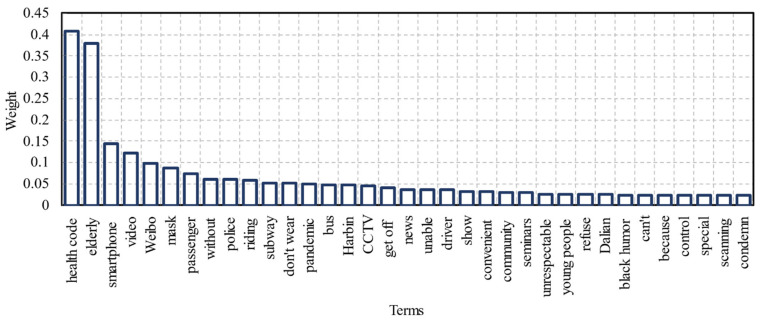
Top 35 most important words in public communication. The weights represent the importance of the terms and the bar with the highest weight indicates that it is the primary focus of public attention.

The top 35 most important terms in the Weibo corpus were extracted from the continuous attention stage after running the TF-IDF algorithm, as shown in Figure 7. The top four terms were “Health Code”, “elderly”, “smartphone”, and “video”, indicating that the majority of Weibo users focused on the news of unfair events. Terms such as “mask”, “pandemic”, and “don’t wear” were also highly weighted, indicating that the public maintained the habit of wearing masks to avoid the virus. This is in line with the pandemic prevention measures within public transportation and most public places that have been propagated by the Chinese government [37]. 

Interestingly, other important terms are also presented in the figure such as “smartphone”, “without”, and “unable”. This shows that the public became aware of the difficulties that elderly people face in obtaining and using health codes through digital devices. Health codes require smartphones and Internet connections. However, the most recent report shows that getting around in China without a smartphone or being off-line is still a difficult task in 2020 [38,39], and this has been a particular problem for the country’s elderly population [40], especially when they travel by public transportation without health codes during the pandemic. Consequently, the general public has tried to speak on behalf of elderly travelers on social media.

Furthermore, the terms “staff”, “driver”, “police”, and “get off” indicate that the public also considered the issues related to mobility services provided by transportation agencies. Moreover, the terms also suggest that the public hopes that people around elderly people (i.e., “passengers” and “young people”) can assist them, rather than ignoring their needs. Elderly people were more likely to learn and use smartphones and the Internet with support from others. Therefore, society, governments, and transportation service providers should work together to help elderly people reap the benefits of the Internet in future situations and eventually improve the equity and fairness of public policies [38].

### 3.3. Discussion Topics

Through the LDA model, the 10,505 messages in the continuous attention stage of the second category were divided into several topics. Then, we inferred the context of the topics based on the given keywords for each topic (see Table 1). In general, there were five categories of public concerns, namely a description of the unfair event, mobility service provided by transportation agencies, status quo of elderly people, pandemic prevention, and government response. Among these, the largest number of messages was on Topic 3, accounting for 33.9% of the total, followed by Topic 2 (33.4%) and Topic 1 (19.8%). The numbers of messages on Topic 4 and Topic 5 were small, accounting for only 12.9%.

As shown in Table 1, Topic 1 mainly refers to when people receive detailed information about the unfair events via news or videos as well as the location where the events occurred. Topic 2 is concerned with the mobility service provided or operated by transportation agencies. After learning about the events, the public also evaluated the service provided in the reported unfair events and offered suggestions to the transportation departments. We list the message examples of Topic 2 in Table 2. Although it is understandable and even necessary for the staff of transportation departments to strictly implement pandemic prevention measures, they are expected to help elderly people when they cannot provide their health code. One such example is how one railway station in Wuxi City in eastern China is helping people without smartphones print out health codes to help them travel [41]. 

Topic 3 concerns people’s reflections on the status quo of elderly travelers and their suggestions for future policy formulations. This is the topic that received the highest attention from the public. The keywords indicate that people have been aware of the difficulties that elderly travelers suffered in the context of the digital era, especially regarding pandemic prevention. Some of the keywords also reflect the public’s suggestions for humane policies and regulations in the transportation system. Example messages in Topic 3 are shown in Table 3. 

Topic 4 focuses on pandemic prevention, with the keywords indicating the significance of the health codes in curbing the virus. Topic 5 is concerned with the governmental response to these unfair events. The keywords indicate that reports of the measures implemented by government agencies dealing with such unfair events were widely spread with the help of the media during the COVID-19 pandemic.

### 3.4. Changes of Weibo Messages on Each Topic

**Figure 8:**
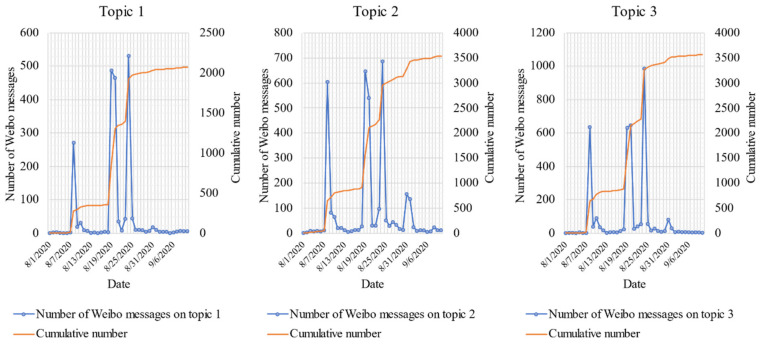
Time series data of public interest in the top three topic categories.

The top three topics were selected to investigate temporal patterns, as shown in Figure 8. At the continuous attention stage, three obvious peaks were noted for public attention on 8 August, 19 August, and 24 August. Based on the observations in Figure 6, the date of these peaks corresponded to the occurrences of a series of unfair events involving elderly travelers. It can be observed that the public’s concern on Topic 1 and Topic 3 started on 8 August and ended on 26 August. However, the number of Weibo messages on Topic 2 fluctuated over a small range of time from 31 August to 2 September. This indicates that after the government’s response to the event, the public was still concerned about the transportation services. Hence, it is suggested that the transportation department update their services and provide feedback to the public. Furthermore, during the continuous attention stage, the public’s concerns on the top three topics lasted for over one month, which was much longer than the average influence duration of a typical event in real-world conditions. We can conclude that public communication over social media can transcend the limitations of time, which allows for the full involvement of social groups in public affairs [20].

### 3.5. Attitude Tendency of the Public

**Figure 9:**
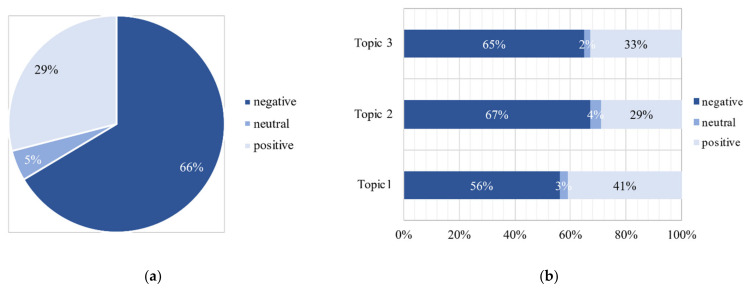
The proportion of attitude tendencies in (**a**) total Weibo messages and the (**b**) top three topics.

Figure 9 presents the proportion of people’s positive, negative, and neutral attitudes toward different topics based on the text sentiment analysis. Overall, as shown in Figure 9a, the percentage of negative attitudes reflected in Weibo messages was high, reaching 66% of the total. This indicates that most of the public holds a negative attitude toward these unfair events concerning elderly people without health codes. As shown in Figure 9b, the level of negative attitudes for Topic 2 was the highest on average, reaching 67% of the total. Messages on Topic 3 were mostly negative, accounting for about 65%. The public was more concerned about the travel status of elderly people while they were not satisfied with the unfair events during the pandemic. This may be related to the fact that the public is more inclined to post negative comments on social media. As Barbalet concluded, negative attitudes are evoked when social norms and values do not match [42]. 

**Figure 10:**
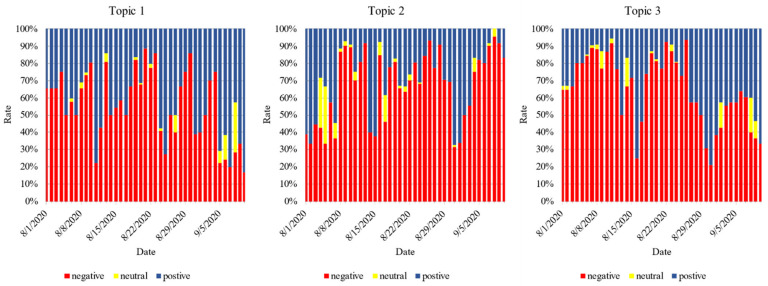
Relative frequencies of attitude tendencies in topics over time.

Figure 10 represents the sentiment proportions for the top three concerns over time. The proportion of negative attitudes toward Topic 1 increased rapidly after the response from the Dalian subway company on 7 August. With a short decline thereafter, there was a long-duration peak again after the Harbin events occurred. Around 24 August, after the government authorities’ response to these events, the proportion of negative attitudes declined rapidly. Since then, the proportion of negative attitudes have remained at a low level. Similar to Topic 1, after the response from the Dalian subway company, the proportion of negative attitudes to Topic 2 also increased rapidly. After a subsequent decline, the proportion increased rapidly with the occurrence of the Harbin event and remained at a high level for about a week. Although the proportion of negative attitudes decreased temporarily after the government’s response to this problem, a rapid increase was observed later, which was much higher than that before the response from the Dalian subway company. Unlike Topic 2, following the government’s response, negative emotions to Topic 3 decreased considerably to a value lower than that at the beginning of the stage, which may be attributed to the increasing public confidence regarding improvements to the status of elderly people. 

The results showed that the proper responses of governmental agencies could reduce the proportion of negative attitudes of the public for a short period, as reflected in the top three topics. Compared with the early days of the continuous attention stage, the proportion of the public’s negative attitudes toward the service by transportation agencies was still very high. Therefore, transportation departments should strengthen staff training and also modify the relevant regulations to avoid the reoccurrence of similar events in the future. 

To improve the equity and fairness of policies, government authorities should strengthen communication and cooperation with transportation agencies to provide a mobility system that will “leave no one behind”. Specifically, governments should first support more alternatives consisting of both traditional and advanced approaches to detect people’s health status, for example, with the use of paper health codes. The government can also build a “health code query platform” involving transportation stakeholders based on individual identities such as the resident identity card (RID) in China. Transportation departments can then design a “no health code channel” for travelers without digital health codes by inquiring about their health status through the platform that is provided by the government. These inclusive and humanized services will help passengers who cannot obtain a digital health code to eliminate technical barriers to ensure their travel and achieve the two objectives of controlling the pandemic and ensuring the daily travel of vulnerable groups.

## 4. Conclusions

During the COVID-19 pandemic, many countries have implemented contact tracing mobile applications to curb the spread of the virus including the health code system launched by the Chinese government. However, due to inadequate consideration of the elderly population, there were a series of unfair events that sparked controversial debates on social media. This study explored the issues concerning elderly travelers without health codes during the COVID-19 pandemic from the perspective of the general public with the help of a dataset of Weibo messages and a proposed hybrid technical framework. The three main findings of this study are as follows:

- Before the spread of sensational information and rumors, the public rarely devoted attention to the problems of elderly travelers on the Internet. A rapid increase in the volume of Weibo messages was observed when the fake news was being reported, indicating that the public is more likely to be influenced by sensational information and rumors during the COVID-19 pandemic. This is the manifestation of the “infodemics” created by social media [43]. When the amount of information excessively increases, rumors and misinformation often begin. Social media can help spread these rumors and misinformation quickly in communities, making it difficult for the public to find trustworthy and reliable guidance during a pandemic. Therefore, it is important to provide timely and reliable information for the public during the COVID-19 pandemic. Social media has become an essential channel for government authorities and service providers to capture people’s demands and to realize better responses when a crisis occurs.
- The top three topics of public concern were extracted including a description of unfair events, mobility service, and their providers, and the status quo of elderly travelers. Furthermore, the duration of the public’s concern on transportation services provision lasted longer than those on the other two topics. Hence, transportation service providers must provide feedback to the public as well as implement improvements in mobility services on time.
- Most people who participated in the discussion expressed negative attitudes regarding these unfair events, with 66% of the attitude tendencies being negative. Although the government released their responses afterward, people remained negative in their comments about transportation departments, while they held a more positive attitude toward the status quo of elderly travelers. Government authorities still need to make a significant effort in promoting the equity and fairness of policies for elderly people. Actively providing affordable training opportunities for information acquisition [7] and improving the design of health codes tailored for the elderly is highly recommended.

Furthermore, based on a seven-month social media dataset, a hybrid NLP proposed approach is applicable for analyzing people’s concerns and response patterns. The results provide insights for governmental authorities and service providers to design an inclusive mobility system, especially for bridging the digital divide for the elderly in the era of ICTs. Our study still has limitations. Due to the sampling bias resulting from the feature of social media platforms, the actual experiences of the elderly people who are not using smartphones may not be accounted for. Other traditional sources of data such as interviews or questionnaires with elderly travelers are necessary to examine the current conclusions and provide an in-depth understanding of the issues facing elderly people.

Recently, the Chinese government began to speed up efforts to meet the nation’s goal of tackling the most pressing issues facing senior citizens. The latest measure released in December 2020 states that health codes shall not be the only certificate to determine whether passengers can use public transportation, and alternative certificates such as valid RIDs, paper certificates for travel, etc. can be adopted [44]. This also demonstrates the influence that public opinion can have on the government’s formulation, improvement, and implementation of public policies. Therefore, the insights proposed in this study using social media data to capture public perceptions toward unfair events are helpful to understand the demands of elderly people in the digital era.

## Figures and Tables

**Table 1:** Recognition results of the latent Dirichlet allocation (LDA) model.

Topic	Meaning of Topic	Keywords	Rate
1	Description of unfair events	video, news, Weibo, Harbin, Dalian, refuse, subway, bus	19.8%
2	Provision of transportation service	driver, police, staff, talk, speak up, blame, patience, explanation, demonstrate	33.4%
3	Status quo of elderly people	society, without, consider, unable to use, smartphones, groups, policies, humanization	33.9%
4	Pandemic prevention	pandemic, prevention, control, personnel, work, requirements, measures, management, implementation, COVID-19	7.3%
5	Government response	CCTV, news, response, support, official, angle, regulation, transportation, discussion	5.6%

**Table 2:** Example messages regarding Topic 2.

	Weibo Messages
1	In principle, the driver is right, but we should think of other ways to help elderly people rather than rudely refuse their access.
2	It is not appropriate for the staff to shout without masks.
3	When observing that elderly people cannot operate smartphones, the staff should offer demonstrations to the elderly people!
4	Understandably, the staff must strengthen the management of pandemic prevention, but their method is not appropriate.
5	Does it mean that an elderly person who cannot use the health code does not deserve to travel by bus? Is it appropriate for the elderly person to be treated unfairly?

**Table 3:** Example messages regarding Topic 3.

Weibo Messages	
1	Using health codes seems convenient, but it does not take into account all citizens, especially elderly people who do not use smartphones.
2	Society is progressing, but people are becoming more selfish. Everybody grows old and we will become old someday.
3	Elderly people are not keeping pace with the times. We should stop to help them.
4	This policy is inconvenient and inhumane because most elderly people cannot use smartphones.
5	Health codes and similar digital products benefit the public, but also damage the ability of elderly groups to enjoy the equity and fairness of society.

## Data Availability

The data presented in this study are available on request from the corresponding author.
